# Environment-Oriented Assessment of Hybrid Methods for Separation of N-Propanol–Water Mixtures: Combination of Distillation and Hydrophilic Pervaporation Processes

**DOI:** 10.3390/membranes15020048

**Published:** 2025-02-05

**Authors:** Huyen Trang Do Thi, Andras Jozsef Toth

**Affiliations:** Department of Chemical and Environmental Process Engineering, Environmental and Process Engineering Research Group, Budapest University of Technology and Economics, Műegyetem rkp. 3, H-1111 Budapest, Hungary; dothihuyentrang@edu.bme.hu

**Keywords:** n-propanol dehydration, distillation, pervaporation, hybrid process, life cycle assessment, decarbonization

## Abstract

This study presents a novel approach to the dehydration of n-propanol using three hybrid methods—D + HPV, D + HPV + D, and D + HPV + D with heat integration—each combining distillation (D) and hydrophilic pervaporation (HPV) without the use of additional solvent agents, as in the most common separation method, extractive distillation. The optimization was performed using a ChemCAD process simulator, targeting 99.9 wt% purity for n-propanol and water. This is the first research to provide a comprehensive cost estimation and carbon footprint analysis for such configurations. Results show the D + HPV + D + HI method provides the best balance of energy efficiency, environmental sustainability, and economic feasibility. It reduced heat duties by 18.5% compared to D + HPV + D, achieved similar CO_2_ emissions to D + HPV with better energy efficiency, and lowered the total annual cost by 37.9% compared to D + HPV. The findings establish D + HPV + D + HI as a promising technology for sustainable and cost-effective n-propanol dehydration.

## 1. Introduction

Pervaporation (PV) is a chemical unit operation in which a mixture is separated by vaporizing it under low pressure on the downstream side of a membrane. Separation occurs through the preferential sorption and diffusion of the desired component across dense membranes. The difference in partial pressures is typically achieved by maintaining a low vapor pressure on the permeate side using a vacuum pump [1,2,3]. Pervaporation is a relatively new technology for separating a wide range of organic–aqueous systems. The growing number of publications, books, and industrial applications highlights the increasing significance of pervaporation as a membrane separation technique [4,5]. Pervaporation offers unique advantages, including high separation efficiency, simple implementation, environmental friendliness, and energy efficiency, which are challenging to achieve with conventional methods. Additionally, pervaporation can be advantageous over distillation due to its lower energy requirements and its ability to separate azeotropic mixtures effectively [3,6].

The pervaporation separation process is governed by selective sorption, dissolution, and diffusion within the membrane. This technique is primarily applied for the dehydration of organic compounds [7,8], the removal of low-concentration organics from aqueous mixtures [9,10], and organic–organic separations [11,12]. Based on the type of permeating component, pervaporation can be classified into two main categories: hydrophilic (HPV) and organophilic pervaporation [3,13].

To remove volatile organic compounds, other separation methods such as distillation, liquid–liquid extraction, carbon absorption, and air stripping are often unsuitable due to feed condition limitations, the production of large volumes of by-products, or the high costs associated with post-treatment processes. In contrast, pervaporation can be applied without these restrictions [14]. Distillation is generally used to remove organic compounds from water. However, it cannot be utilized for thermally sensitive organic compounds. Additionally, as noted by Fleming and Slater [15,16], pervaporation offers several advantages over traditional distillation: lower energy consumption, since only the permeate fraction of the liquid is vaporized (I), lower operating temperatures, making it suitable for heat-sensitive compounds (II), the ability to separate azeotropes (III), and the elimination of the need for additional components, as required in extractive or azeotropic distillation processes (IV) [3,17]. These benefits—combined with relatively mild operating conditions and its high separation efficiency—make pervaporation an excellent choice for such separations [14,18,19].

There are numerous publications in the literature dealing with the dehydration of isopropanol [20,21,22]; however, the dehydration of n-propanol is a less researched area. The dehydration of n-propanol is a crucial reaction in organic chemistry and industrial applications, as it provides an efficient route to producing propene, a key building block in polymer and petrochemical industries. This reaction is significant in developing catalytic processes, particularly in optimizing acid catalysts such as alumina and zeolites for selective and energy-efficient conversions. From a sustainability perspective, bio-based n-propanol derived from renewable sources can be dehydrated to propene, reducing dependence on fossil fuels and supporting green chemistry initiatives [23]. Studying n-propanol dehydration contributes to fundamental research and industrial advancements, enabling more efficient and environmentally friendly chemical production.

Toth [24] summarized the main research works about n-propanol dehydration and it was determined that mostly polymer membranes are used. The total flux values are typically between 0.08 and 8.8 kg/m^2^h. Toutianoush et al. [25] reached the highest separation factor value (~6000) and pervaporation separation index value (~7200) using polyvinylamine/polyvinylsulphate membrane. Sokolova et al. [26] notes the highest total flux value (~8.8 kg/m^2^h) with a poly(urethane-imide)–PUI-2000-type membrane. Further descriptive data about n-propanol dehydration membranes can be found in the work of Toth [24].

In the available literature [27,28,29], successful separation is typically achieved by extractive distillation using glycerol as the entrainer. Wang et al. [28] introduced energy-efficient distillation and pervaporation techniques for separating a ternary mixture of n-propanol, acetonitrile, and water. Their recommended method also relies on extractive distillation with glycerol as the entrainer. Similarly, Wu et al. [27] proposed a hybrid method combining extractive distillation and pervaporation for dehydrating n-propanol. Their approach proved to be both efficient and cost-effective, achieving product purities of up to 99.9% with glycerol as the extractive agent.

Toth [24] developed the pervaporation-based separation method for n-propanol dehydration. The present work focuses on further developing the previously presented distillation-membrane process. The main objectives are the optimization (1), the environment-oriented assessment (2), and the cost estimation (3) of the different n-propanol dehydration methods. The combination of methods is necessary because of the azeotrope, or there is a relatively small amount of n-propanol in the feed stream which would require a very large pervaporator if the separation would be performed in one step.

## 2. Materials and Methods

Pervaporation is recognized as a competitive alternative to distillation in certain separation processes [6,24]. This study aims to explore the separation of an n-propanol–water mixture using a hybrid distillation and hydrophilic pervaporation approach within a professional simulation environment.

The first step is to define the problem and establish the objectives. For hybrid systems, minimizing the membrane size is desirable for economic efficiency. To achieve this, distillation must produce the highest possible purity [30]. Accordingly, the azeotropic mixture of 70.8 wt% n-propanol and 29.2 wt% water is subjected to hydrophilic pervaporation, targeting a final water and n-propanol purity of 99.9 wt%.

In this study, the semiempirical pervaporation model developed by Szilágyi and Tóth [31] is utilized. Parameter estimation, based on experimental data, is necessary to determine the model parameters. Following this, the parameters are validated by comparing experimental results with those predicted by the model. Once the parameters are deemed reliable, detailed modeling is carried out in a flowsheet simulation environment using the ChemCAD 6.5.6 software for the n-propanol dehydration process.

The first phase of the simulation process involves validating the model under experimental conditions. The flowsheet software evaluates the model’s accuracy by comparing simulated results with experimental data. If the validation is successful, the optimization phase can begin. For the n-propanol–water separation process, optimization focuses on determining the effective membrane surface area (A) required for the separation [32].

### 2.1. Modeling of Pervaporation

Numerous models have been proposed in the literature to describe pervaporation transport processes, including the total solvent volume ratio model, the pore-flow model, and the solution–diffusion model [19,33,34]. In this study, the hydrophilic PV model developed by Szilágyi and Tóth [31] was applied, which is expressed as:

(1) $$J_{i}=\frac{1}{1+\left\{\frac{\left[{\bar{D}}_{i}\cdot exp\left({x_{i1}}^{B}\right)\right]}{\left({Q_{0}\cdot p}_{i0}\cdot {\bar{\gamma }}_{i}\right)}\right\}}\cdot \frac{\left[{\bar{D}}_{i}\cdot exp\left({x_{i1}}^{B}\right)\right]}{{\bar{\gamma }}_{i}}\cdot \left(\frac{p_{i1}-p_{i3}}{p_{i0}}\right)\;i=\left(1,\ldots ,k\right)$$

This pervaporation model is based on the frameworks established by Rautenbach [35] and Valentinyi et al. [4], with modifications to account for the concentration dependence of the transport coefficient ($\bar{D_{i}}$) and the temperature dependence of the process [36]. The permeability coefficient (Q_0_) of the porous support layer is assumed to be significantly larger than $D_{i}$, rendering its resistance negligible. As a result, the initial terms in Equation (1) can be disregarded [36], resulting in simplified models that are more practical for calculations.

(2) $$J_{i}=\frac{{\bar{D}}_{i}\cdot exp\left({x_{i1}}^{B}\right)}{{\bar{\gamma }}_{i}}\cdot \left(\frac{p_{i1}-p_{i3}}{p_{i0}}\right)$$

The partial pressures ($p_{i0}$) were calculated using the Antoine equation [32]:

(3) $$p_{i0}\;=e^{\left[A+\frac{B}{T}+ClnT+DT^{E}\;\right]}\cdot 10^{-5}$$

here, there were A–E material-specific constants. The temperature dependence of the transport coefficient is described by an Arrhenius-type equation:

(4) $$\bar{D_{i}}=\bar{{D_{i}}^{*}}\cdot e^{\frac{E_{i}}{R}\left(\frac{1}{T^{*}}-\frac{1}{T}\right)}$$

$E_{i}$ represents the activation energy of component i, associated with the transport coefficient, while $T^{*}$ denotes the reference temperature (293 K in Equation (4)). Activity coefficients are determined using the Wilson equations, with detailed formulas for the binary system provided in the Supporting Information.

The activation energies, transport coefficients, and B parameters in Equation (2) for both components in the pervaporation model were derived from experimental data using a non-linear regression method implemented in STATISTICA^®^ 14 software. The accuracy of the model was evaluated by minimizing the discrepancy between the measured and predicted values using an objective function (OF) in Equation (5).

(5) $$OF=\sum_{i=1}^{n}{\left(\frac{J_{i,measured}-J_{i,modelled}}{J_{i,measured}}\right)}^{2}$$

The calculated coefficients can be seen in Table 1 in the case of the PERVAP™ 1201 composite hydrophilic pervaporation membrane [24].

### 2.2. Simulation of Hybrid Distillation and Pervaporation Methods

The goal was to separate a 10 wt% n-propanol–90 wt% water mixture with a stream of 1000 kg/h at 20 °C feed temperature and 1 bar feed pressure. The objective was to obtain n-propanol and water products with a purity of 99.9 wt%. ChemCAD V6.5, a professional process simulation software, was used to model the hybrid separation process. Three different configurations were investigated. The simulation model was pre-validated in experimental conditions [24].

#### 2.2.1. Hybrid Distillation–Hydrophilic Pervaporation Method (D + HPV) for N-Propanol and Water Purification (Configuration “A”)

**Figure 1:**
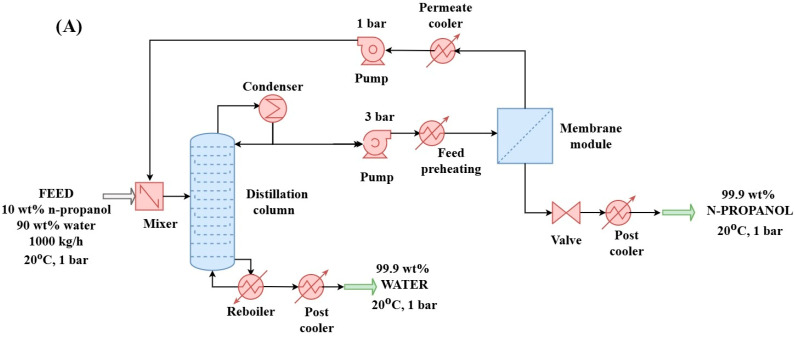
Distillation + hydrophilic pervaporation method (configuration “A”) for separation of n-propanol–water binary mixture in flowsheet environment.

The hybrid process consists of distillation and pervaporation dehydration steps, as illustrated in Figure 1. In the first step, the distillation column was optimized with 40 theoretical stages, with the feed introduced at the 20th stage. Water was removed from the bottom of the column, while the n-propanol phase (distillate product in azeotropic composition) was directed to the pervaporation membrane. High-purity n-propanol was recovered as the retentate stream (R) from the pervaporation process. The distillation step was performed using the UNIQUAC thermodynamic model, with the column operating at a pressure of 1 bar. Heat exchangers maintained the final products at 20 °C.

**Figure 2:**
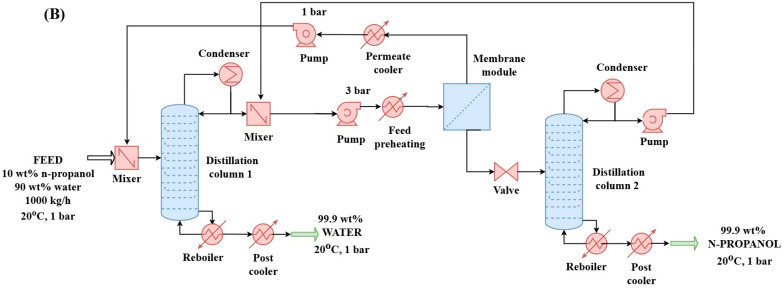
Distillation + hydrophilic pervaporation + distillation method (configuration “B”) for separation of n-propanol–water binary mixture in flowsheet environment.

**Figure 3:**
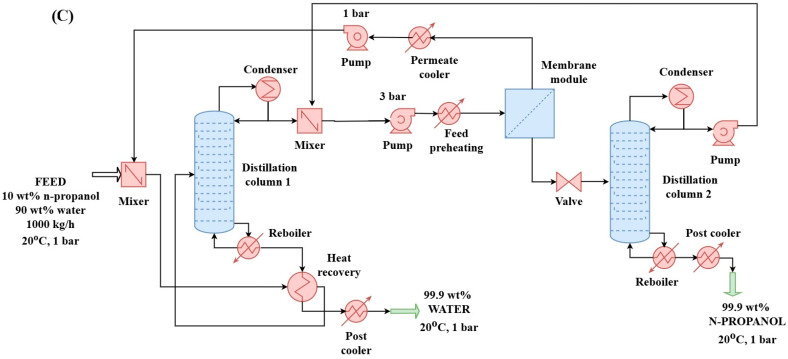
Distillation + hydrophilic pervaporation + distillation with heat integration (HI) method (configuration “C”) for separation of the n-propanol–water binary mixture in a flowsheet environment.

During the pervaporation step, the liquid feed passed through a membrane driven by a partial pressure gradient. The stream passing through the membrane was collected as the permeate stream (P), while the remaining portion was the retentate (R). Vacuum pressure was applied on the permeate side to enhance separation efficiency. The permeate product exited from the membrane modules in vapor phase. The permeate cooler part was used to bring it back to a liquid phase. The pervaporation modules were arranged in series to increase the mass flow rate incrementally, with heat exchangers ensuring the appropriate temperature at each stage. Only one pervaporation module can be seen in Figure 1, Figure 2 and Figure 3. The structure layout can be studied in Figure S1 in the Supporting Information.

#### 2.2.2. Hybrid Distillation–Hydrophilic Pervaporation–Distillation Method (D + HPV + D) for N-Propanol and Water Purification (Configuration “B”)

In the D + HPV + D process, the pervaporation step proceeded beyond the azeotropic point. A secondary distillation column was then introduced, where the top product had an azeotropic composition, while the bottom product achieved high purity (see Figure 2). The final retentate flow from the hydrophilic pervaporation was directed into the second distillation column. If the pressure was too high, a valve reduced it before the inlet. Both distillation columns operated at a pressure of 1 bar. A thorough optimization of both the pervaporation system and the second distillation column ensured stable operation and high-quality product output. The distillate product stream from the second column was recycled back after the first column.

#### 2.2.3. Hybrid Distillation–Hydrophilic Pervaporation–Distillation Method with Partial Heat Integration (D + HPV + D + HI) for N-Propanol and Water Purification (Configuration “C”)

The hybrid distillation and pervaporation process require a considerable amount of heat, so heat integration was incorporated into the analysis. Additionally, excess heat was redirected to the necessary points to optimize energy consumption. In the partial heat integration shown in Figure 3, the heat from the bottom product of the first distillation column was used to preheat the incoming feed. An alternative configuration could involve using heat from the product stream of the second column; however, due to the small temperature difference, this was not considered for the second distillation column.

### 2.3. Life Cycle Assessment

Environmental protection is increasingly central to daily life, driving the need for tools to evaluate and mitigate environmental impacts. Life cycle assessment (LCA) is a widely used method that examines a product’s entire life cycle from raw material extraction to disposal, assessing impacts like greenhouse gas (GHG) emissions and energy use [37]. LCA supports sustainable decisions by comparing processes, materials, and products, though it faces challenges such as costly data collection, subjective impact weighting, and limited consideration of long-term use [38]. This study follows LCA guidelines from ISO 14040:2006 [39] and ISO 14044:2006 [40], encompassing four phases: goal and scope definition, inventory analysis, impact assessment, and result interpretation [41].

#### 2.3.1. Goal and Scope

The environmental impact of three hybrid technologies—D + PV, D + PV + D, and D + PV + D + HI—for separating 10 wt% n-propanol–90 wt% water binary mixtures was evaluated using LCA. The analysis employed the Ecoinvent V3.9.1 database, SimaPro V9.5 software, and the environmental footprint (EF) V3.1 method. A “gate-to-gate” approach focused on the operational phase, with a functional unit (FU) of 1 kg of 99.9 wt% purified n-propanol per hour of operation.

#### 2.3.2. Inventory Data

This study used a “gate-to-gate” analysis to evaluate n-propanol–water separation using hybrid technologies, assessing material and energy requirements for recovering purified components. Heat was supplied either through district heating systems or industrial sources powered by natural gas. The geographical scope of this study was considered to be global. Data for producing 1 kg of 99.9 wt% pure n-propanol, derived from ChemCAD simulations, is summarized in Table 2.

#### 2.3.3. Life Cycle Impact Assessment

The goal of LCIA is to understand the environmental impacts of life cycle inventory results, focusing on human health, ecosystems, and resources. This study summarizes environmental impact as an environmental or carbon footprint using the adapted environmental footprint (EF) V3.1 method, which includes 28 impact categories. Key areas analyzed are climate change (kg CO_2_ equivalent over 100 years), human carcinogenic toxicity (CTUh—cases per kilogram of substance emitted per unit mass), and ecotoxicity (CTUe, PAF.m^3^.year/kg).

## 3. Results and Discussion

### 3.1. Optimization of the Hybrid Methods for N-Propanol–Water Separation

The detailed simulation results are provided in Table 3 and Table 4. The goal was to enhance the efficiency of the D + HPV and D + PV + D processes by recovering 99.9 wt% purified n-propanol and water using a process simulator supported by ChemCAD software.

The “Water” and “N-propanol” product streams are in bold in Table 3 and Table 4. It is important to note that achieving a 99.9 wt% n-propanol concentration requires an increase in the pervaporation membrane area by adding additional membrane units. The effectiveness of applying the double distillation column is demonstrated, because in these cases the centrally located membrane process requires significantly smaller dimensions to reach the appropriate separation goals. The calculated heat duties of the three different hybrid n-propanol dehydration methods can be seen in Table 5 and Table 6.

The results show that the heating demand of the first distillation column can be significantly reduced by using heat integration (HI). This is particularly important because the highest heat demand of the processes is in the distillation columns. The retentate heating values are lower in the second and third cases because there is a lower necessary total membrane area in the case of the D + HPV + D and D + HPV + D + HI methods. The sum of heating and cooling duties must be equal to each other. This is clearly correct in Table 5 and Table 6.

### 3.2. Life Cycle Assessment of Hybrid Methods for N-Propanol Dehydration in Case Obtaining 1 kg of Products with a 99.9 wt% Purity

The detailed results of the LCA, based on the EF v3.1 (adapted) method, are presented in Table S1 of the Supporting Information. It is worth noting that heat in all configurations is supplied by natural gas.

Among the evaluated technologies, D + HPV demonstrates the lowest impacts on climate change, fossil resource depletion, ecotoxicity, and human toxicity. This is primarily due to its minimal carbon footprint and efficient energy consumption. In contrast, the D + HPV + D configuration shows the highest impacts across nearly all categories, with significant increases in climate change, water use, and fossil resource depletion, driven by the energy-intensive second distillation process. The D + HPV + D + HI configuration acts as an intermediate option, offering slightly lower impacts in specific categories (e.g., climate change and fossil resource use) than D + HPV + D but suffering from substantially higher water consumption, reducing its overall benefits.

Regarding CO₂ emissions, the D + HPV method has the lowest value at 0.957 kg CO₂-eq, followed closely by D + HPV + D + HI with 0.965 kg CO_2_-eq. These two configurations show very similar impacts, differing by only 0.8%. On the other hand, the D + HPV + D method exhibits the highest emissions at 1.03 kg CO₂-eq, which is 7.1% higher than D + HPV, indicating a greater contribution to global warming.

**Figure 4:**
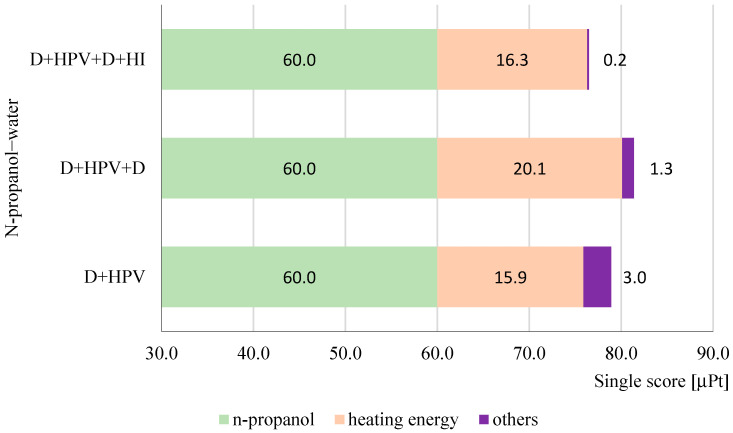
The process’ contribution to the single score for the investigated n-propanol–water separation technologies.

Based on the process contribution to the total single score in Figure 4, the ranking of these three technologies is as follows: D + HPV + D + HI (76.5 μPt), D + HPV (78.9 μPt), and D + HPV + D (81.4 μPt). Across all configurations, the n-propanol contribution remains constant at 60.0 μPt, indicating that the environmental impact of n-propanol production or use is independent of the process configuration. However, n-propanol consistently accounts for the majority of the environmental impacts, contributing 76.0%, 73.7%, and 78.4% of the total single score for D + HPV, D + HPV + D, and D + HPV + D + HI, respectively. This high percentage underscores the substantial environmental burden of n-propanol production or separation, likely due to resource-intensive processes such as energy consumption, raw material use, and emissions.

Heat, the second-largest contributor, plays a crucial role in differentiating the environmental impacts of these configurations. D + HPV (15.9 μPt) demonstrates the lowest heat-related impact, underscoring its lowest energy requirement. In contrast, D + HPV + D (20.1 μPt) shows a 26.4% increase due to the additional energy demands of the second distillation step. D + HPV + D + HI (16.3 μPt) reflects only a modest 2.5% increase compared to D + HPV, balancing slightly higher energy needs with lower overall impacts than D + HPV + D.

Overall, the D + HPV + D + HI configuration emerges as the most balanced choice, with the lowest overall environmental impact despite slightly higher heat-related contributions compared to D + HPV. Further optimizing heat efficiency in this configuration could make it the most sustainable option for n-propanol–water separation.

Replacing fossil-based energy sources (e.g., natural gas) in the D + HPV + D + HI system with renewables like solar energy and biofuel reduces fossil CO₂ emissions. Switching to biofuel (biomethane) reduces emissions by 18.7% (0.785 kg CO_2_-eq), while solar energy achieves a 27.8% reduction (0.697 kg CO_2_-eq). The most sustainable option is heating reuse from municipal untreated waste wood incineration, which has the lowest impact at 0.668 kg CO_2_-eq—a 30.8% reduction compared to natural gas and 4.2% lower than solar energy. This highlights the significant potential for emission reductions through renewable or repurposed energy sources.

### 3.3. Economic Results of Hybrid Separation Methods for N-Propanol–Water Separation

The total annual cost (TAC) encompasses both the total investment costs (TIC) for all units and the total operating costs (TOC) for utilities. In this analysis, the distillation column and pervaporation membrane are assumed to have lifespans of 10 years and 2 years, respectively, with 8000 h of operation per year. Utility costs are 4.43 EUR/GJ for chilled water at 278 K and 14.05 EUR/GJ for low-pressure steam at 433 K [42]. For the PV membranes, the investment cost and the replacement cost were also calculated [43]. The cost calculation equations with correction factors, based on Douglas [44] and González et al. [43], are provided in Table S2 in the Supporting Information.

**Figure 5:**
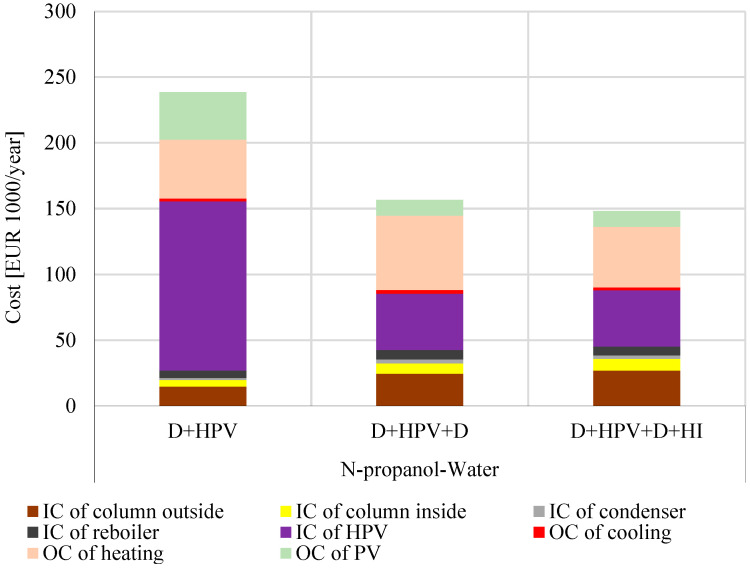
The total investment costs (TIC) and the total operation costs (TOC) of the investigated hybrid methods for n-propanol–water separation: values in 1000 EUR/year.

**Figure 6:**
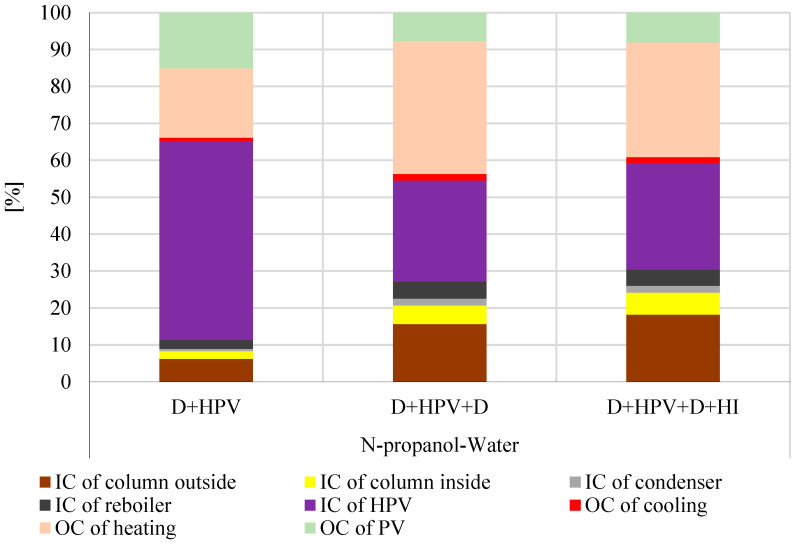
The total investment costs (TIC) and the total operation costs (TOC) of the investigated hybrid methods for n-propanol–water separation: percentages.

Figure 5 provides a comprehensive comparison of the total costs for the hybrid separation systems examined, while Figure 6 shows the percentage contributions of TIC and TOC to the TAC for each process. Further details on the components of TOC, TIC, and TAC, such as distillation columns, pervaporation membranes, and heat exchangers, are available in Tables S3–S5 in the Supporting Information.

As shown in Figure 5, the D + HPV method has the highest TIC (156,000 EUR/year), primarily due to the substantial investment required for the hydrophilic pervaporation membranes. This procedure also has the highest TOC (83,000 EUR/year, making it even more justified to develop further hybrid separation procedures. In comparison, the D + HPV + D method lowers the TIC (85,000 EUR/year) by reducing hydrophilic pervaporation membrane costs and the TOC (71,000 EUR/year) can also be reduced. The D + HPV + D + HI method proved to be the best solution because the TOC (60,000 EUR/year) can be reduced a little further, while there is no significant increase in the TIC (88,000 EUR/year) value. Therefore, it can be stated that in terms of the total annual cost (148,000 EUR/year), the D + HPV + D + HI procedure proved to be the best, followed closely by the D + HPV + D procedure (TAC:157,000 EUR /year), and finally, D + HPV is significantly less favorable (TAC: 239,000 EUR/year).

According to Figure 6, the D + HPV method allocates the largest portion of the investment (54%) to the hydrophilic pervaporation system, with smaller contributions from the external column side (6%) and the internal column side (2%). Conversely, the D + HPV + D and D + HPV + D + HI methods reduce the investment cost of the pervaporation system’s share (27% and 29%, respectively) by redistributing costs to the external column side (16% and 5%, respectively) and the internal column side (18% and 6%, respectively), resulting in a more balanced TIC distribution. In terms of operating costs, the D + HPV method achieves the lowest share (35%), driven by reduced heating costs (19%) and relatively higher pervaporation costs (15%). Meanwhile, the D + HPV + D method incurs the highest TOC (45%), mainly due to increased heating expenses (36%), while cooling and the membrane costs remain minimal (10%). The D + HPV + D + HI method balances TOC at 41% by slightly reducing heating costs (31%) while keeping cooling and the membrane shares consistent (10%).

Overall, it can be said that the most effective reduction in total annual cost can be achieved by reducing the membrane size and the heating demand. The trends described above for hybrid procedures are consistent with previous literature findings [6,45]. The cost estimation of Wu et al. [27] also demonstrates the effectiveness of adding pervaporation for the separation task of n-propanol dehydration. According to their calculations, integrating pervaporation with extractive distillation using glycerol can lower the total annual cost by over 20%.

## 4. Conclusions

This study introduced and evaluated three innovative hybrid configurations for n-propanol dehydration: D + HPV, D + HPV + D, and D + HPV + D + HI. These methods combine distillation with hydrophilic pervaporation in a binary separation process, uniquely avoiding additional separating agents. For the first time, comprehensive cost estimation and carbon footprint analyses were conducted for such systems, offering fresh insights into their feasibility and environmental impact.

Key findings include:

- Process efficiency: The D + HPV + D + HI configuration demonstrated superior energy efficiency, reducing the calculated heat duties of the D + HPV + D by 18.5% through heat integration.
- Environmental sustainability: While the D + HPV method achieved the lowest carbon footprint (0.957 kg CO₂-eq), the D + HPV + D + HI method offered a balanced approach, emitting 0.965 kg CO₂-eq—a difference of just 0.8%—while maintaining high energy efficiency.
- Economic feasibility: The D + HPV + D + HI configuration achieved the lowest total annual cost (148,000 EUR/year), outperforming the other methods by optimizing heat utilization and minimizing membrane area requirements.

These findings underscore the novelty and practicality of these hybrid processes as viable alternatives for n-propanol dehydration. Integrating renewable and repurposed energy sources could enhance these systems’ environmental and economic performance. This research lays a solid foundation for broader applications of hybrid separation technologies in solvent dehydration, paving the way for more sustainable industrial practices.

The D + HPV + D + HI method has also proven effective in the case of isobutanol–water separation [45]. It is important to mention that detailed energetic and environmental studies should always be performed for the given dehydration case. In the case of distillation-based processes, the heating requirement is a significant factor in operating cost, energy consumption, and environmental impact. By reducing the heating requirement using hybrid processes based on thermal integration, the extent of the listed effects can be reduced compared to traditional separation techniques, such as distillation. Thus, the D + HPV + D + HI process has several advantages.

## Figures and Tables

**Table 1:** Activation energies, transport coefficients, B constant parameters and objective functions estimated by STATISTICA^®^ software [24].

	n-Propanol	Water
$E_{i}$ [kJ/kmol]	29,966	27,707
${\bar{D}}_{i}$ [kmol/m^2^h]	2.32 × 10^−3^	2.40 × 10^−5^
B [-]	−12.08	8.38
OF [-]	0.125	0.123

**Table 2:** Inventory data of LCA.

		D + HPV	D + HPV + D	D + HPV + D + HI
Input	n-propanol [kg]	1.01	1.01	1.01
	Water [kg]	9.07	9.07	9.07
	Heating energy [MJ]	7.62	9.61	7.83
	Cooling water [L]	8.85	14.07	17.37
Output	n-propanol [kg]	1.00	1.00	1.00
	Water [kg]	9.08	9.08	9.08
	Cooling water [L]	8.85	14.07	17.37

**Table 3:** Optimized results of the investigated hybrid methods—first distillation column.

Parameters		Unit	n-Propanol–Water Binary Mixture		
			D + HPV	D + HPV + D	D + HPV + D + HI
First distillation column	Total plates	-	40	40	40
	Feed plate	-	20	20	20
	Reflux ratio	-	1	1	1
	Distillate product stream	kg/h	140.9	140.3	140.3
	Distillate product temperature	°C	87.0	87.0	87.0
	Alcohol of distillate product	wt%	70.8	70.8	70.8
	Bottom product stream	kg/h	900.8	900.8	900.8
	Bottom product temperature	°C	99.4	99.4	99.4
	Alcohol of bottom product	wt%	0.1	0.1	0.1
	Reboiler duty	MJ/h	664.7	663.1	363.7
	Condenser duty	MJ/h	−330.1	−328.9	−328.9

**Table 4:** Optimized results of the investigated hybrid methods—second distillation column and pervaporation unit.

Parameters		Unit	n-Propanol–Water Binary Mixture		
			D + HPV	D + HPV + D	D + HPV + D + HI
Second distillation column	Total plates	-	-	30	30
	Feed plate	-	-	15	15
	Reflux ratio	-	-	3	5
	Distillate product stream	kg/h	-	43.8	43.8
	Distillate product temperature	°C	-	87.0	87.0
	Alcohol of distillate product	wt%	-	70.8	70.8
	Bottom product stream	kg/h	-	99.2	99.2
	Bottom product temperature	°C	-	96.7	96.7
	Alcohol of bottom product	wt%	-	99.9	99.9
	Reboiler duty	MJ/h	-	206.4	329.4
	Condenser duty	MJ/h	-	−196.0	−319.0
HPV	Number of module units	piece	10	6	6
	Total membrane area	m^2^	360	120	120
	Permeate product stream	kg/h	41.6	41.2	41.2
	Retentate product stream	kg/h	99.2	143.0	143.0
	Retentate product heating	MJ/h	90.0	81.7	81.7
	Permeate cooler	MJ/h	−108.8	−107.8	−107.8

**Table 5:** Calculated heat duty values of the three different hybrid n-propanol dehydration methods.

Calculated Heat Duties [MJ/h]		D + HPV		D + HPV + D		D + HPV + D + HI	
		Q_heating_	Q_cooling_	Q_heating_	Q_cooling_	Q_heating_	Q_cooling_
Distillation column 1	Reboiler	664.7		663.1		363.7	
	Condenser		−330.1		−328.9		−328.9
	Post-cooler		−299.5		−299.5		
Hydrophilic pervaporation	Feed preheating	1.4		1.9		1.9	
	Retentate heating	90.0		81.7		81.7	
	Permeate cooler		−108.8		−107.8		−107.8
	Post-cooler		−17.7				
Distillation column 2	Reboiler			206.4		329.4	
	Condenser				−196.0		−319.0
	Post-cooler				−21.0		−21.0
Total		756	−756	953	−953	777	−777

**Table 6:** Calculated heat duty percentages of the three different hybrid n-propanol dehydration methods.

Calculated Heat Duties [%]		D + HPV		D + HPV + D		D + HPV + D + HI	
		Q_heating_	Q_cooling_	Q_heating_	Q_cooling_	Q_heating_	Q_cooling_
Distillation column 1	Reboiler	87.9		69.6		46.8	
	Condenser		43.7		34.5		42.4
	Post-cooler		39.6		31.4		
Hydrophilic pervaporation	Feed preheating	0.2		0.2		0.2	
	Retentate heating	11.9		8.6		10.5	
	Permeate cooler		14.4		11.3		13.9
	Post-cooler		2.3				
Distillation column 2	Reboiler			21.7		42.4	
	Condenser				20.6		41.1
	Post-cooler				2.2		2.7
Total		100	100	100	100	100	100

## Data Availability

The original contributions presented in this study are included in the article/Supplementary Materials. Further inquiries can be directed to the corresponding author.

## Supplementary Materials

The following supporting information can be downloaded at: https://www.mdpi.com/article/10.3390/membranes15020048/s1, Figure S1: The structure layout of pervaporation modules. Table S1: The results of LCA based on EF V3.1 (adapted) method; Table S2 [46]: The cost calculation of hybrid process; Table S3: TIC, TOC, TAC of the distillation columns of the investigated hybrid methods; Table S4: TIC, TOC, TAC of the pervaporation membranes of the investigated hybrid methods; Table S5: TIC, TOC, TAC of the heat exchangers of the investigated hybrid methods.

## Nomenclature

A	Membrane transfer area	$\left[m^{2}\right]$
B	Constant in Equations (1) and (2)	$\left[-\right]$
D	Distillate product, distillation	
${\bar{D}}_{i}$	Transport coefficient of component i	$\left[kmol/\left(m^{2}h\right)\right]$
${\bar{D}}_{i}^{*}$	Relative transport coefficient of component i	$\left[kmol/\left(m^{2}h\right)\right]$
$E_{i}$	Activation energy of component i in Equation (4) for temperature dependence of the transport coefficient	$\left[kJ/mol\right]$
F	Feed	
i	Component number	
j	Component number	
$J_{i}$	Partial flux	$\left[kg/\left(m^{2}h\right)\right]$
N	Number of theoretical stages	[-]
P	Permeate	
$p_{i0}$	Pure *i* component vapor pressure	$\left[bar\right]$
$p_{i1}$	Partial pressure of component i on the	
	liquid phase membrane side	$\left[bar\right]$
$p_{i3}$	Partial pressure of component i on the	
	vapor phase membrane side	$\left[bar\right]$
$p_{3}$	Pressure on the permeate side	$\left[bar\right]$
$Q^{*}$	Heat stream	$\left[MJ/h\right]$
Q_0_	Permeability of the porous supporting layer	
	of the membrane	[$kmol/\left(m^{2}hbar\right)$]
R	Retentate	
Ʀ	Gas constant	$\left[kJ/\left(kmolK\right)\right]$
T	Temperature	$\left[℃\right]$
$T^{*}$	Reference temperature: 293 K	
$x_{i1}$	Concentration of component i in the feed	$\left[wt\%\right]$
*Abbreviations*		
CTUe	Comparative toxic unit for ecosystem	
CTUh	Comparative toxic unit for human	
EF	Environmental footprint	
FU	Functional unit	
GHG	Greenhouse gas	
HI	Heat integration	
HPV	Hydrophilic pervaporation	
hydr	Hydrophilic	
IC	Investment cost [EUR]	
LCA	Life cycle assessment	
LCI	Life cycle inventory	
LCIA	Life cycle inventory assessment	
OC	Operating cost [EUR]	
OF	Objective function	
PAF	Potentially affected fraction	
PV	Pervaporation	
TAC	Total annual cost [EUR/year]	
TIC	Total investment cost [EUR/year]	
TOC	Total operation cost [EUR/year]	
*Greek letters*		
${\bar{\gamma }}_{i}$	Average activity coefficient of component i	
$\gamma_{i1}$	Activity coefficient of component i in the feed	
δ	Membrane thickness	$\left[\mu m\right]$
∆T	Temperature difference	$\left[K\right]$
