# Supplementary material for: Environment-Oriented Assessment of Hybrid Methods for Separation of N-Propanol–Water Mixtures: Combination of Distillation and Hydrophilic Pervaporation Processes
Source: Membranes (Basel). 2025 Feb 5;15(2):48. doi: 10.3390/membranes15020048 (PMC11857795; doi:10.3390/membranes15020048)
Supplement: Supplementary file 1 [file membranes-15-00048-s001.zip › membranes-3426268-supplementary.pdf]

Supporting Information

Environment-oriented Assessment of Hybrid Methods for Separation of N-propanol-Water Mixtures: Combination of Distillation and Hydrophilic Pervaporation Processes

Huyen Trang Do Thi[1], Andras Jozsef Toth[1,*]

[1] Environmental and Process Engineering Research Group, Department of Chemical and Environmental Process Engineering, Budapest University of Technology and Economics, H-1111, Hungary, Budapest, Műegyetem rkp. 3.

* Corresponding author. E-mail address: andrasjozseftoth@edu.bme.hu, Tel: +36 1 463 1490; Fax: +36 1 463 3197, ORCID: 0000-0002-5787-8557

Binary Wilson Activity Model [1]

For the component *i*, the feed side activity coefficient is:

$$\ln\gamma_{i1} = \ln\left[x_{i1} + \Lambda_{ij} \cdot (1 - x_{i1})\right] + (1 - x_{i1}) \cdot \left[\frac{\Lambda_{ij}}{x_{i1} + \Lambda_{ij} \cdot (1 - x_{i1})} - \frac{\Lambda_{ji}}{(1 - x_{i1}) + \Lambda_{ji} \cdot x_{i1}}\right] \quad \text{(S1)}$$

where the Λ_{ij} and Λ_{ji} coefficients were obtained by the following formulas:

$$\Lambda_{ij} = \frac{V_j}{V_i} \cdot \exp\left(-\frac{A_{ij}}{RT}\right) \qquad \Lambda_{ji} = \frac{V_i}{V_j} \cdot \exp\left(-\frac{A_{ji}}{RT}\right) \quad \text{(S2)}$$

where the V_i and V_j are the molar volume of pure liquid, and it was calculated as follows:

$$V_i = \frac{B_i^{\left[1+\left(1-\frac{T}{C_i}\right)^{D_i}\right]}}{A_i} \qquad V_j = \frac{B_j^{\left[1+\left(1-\frac{T}{C_j}\right)^{D_j}\right]}}{A_j} \quad \text{(S3)}$$

where *A*, *B*, *C* and *D* are the constants of the Antoine equation for the *i* and *j* components, respectively.

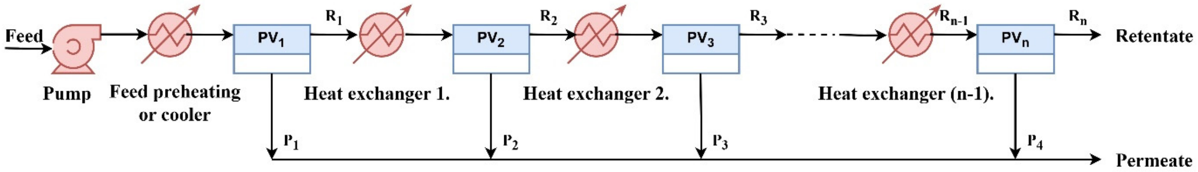

Figure S1. The structure layout of pervaporation modules.

Table S1. The results of LCA based on EF V3.1 (adapted) method.

Impact category	Unit	D+HPV	D+HPV+D	D+HPV+D+HI
Ecotoxicity, freshwater-part 1	CTUe	1.80E+01	1.80E+01	1.80E+01
Ecotoxicity, freshwater-part 2	CTUe	3.76E+00	3.78E+00	3.77E+00
Ecotoxicity, freshwater-inorganic	CTUe	5.12E+00	5.16E+00	5.13E+00
Ecotoxicity, freshwater-organic-p.1	CTUe	1.66E+01	1.66E+01	1.66E+01
Ecotoxicity, freshwater-organic-p.2	CTUh	7.79E-02	7.95E-02	7.81E-02
Human toxicity, cancer	CTUh	2.64E-10	2.74E-10	2.65E-10
Human toxicity, cancer-inorganic	CTUh	1.77E-10	1.82E-10	1.78E-10
Human toxicity, cancer-organic	CTUh	8.69E-11	9.23E-11	8.74E-11
Human toxicity, non-cancer	CTUh	1.34E-08	1.35E-08	1.34E-08
Human toxicity, cancer-inorganic	CTUh	7.51E-09	7.62E-09	7.52E-09
Human toxicity, cancer-organic	CTUh	5.86E-09	5.87E-09	5.86E-09
Particulate	disease incidence	3.31E-08	3.34E-08	3.31E-08
Ionizing radiation	kBq U-235-eq	1.22E-02	1.23E-02	1.22E-02
Ozone depletion	kg CFC11-eq	1.34E-08	1.47E-08	1.35E-08
Climate change	kg CO_2-eq	9.57E-01	1.03E+00	9.65E-01
Climate change- Biogenic	kg CO_2-eq	6.99E-04	7.07E-04	7.00E-04
Climate change- Fossil	kg CO_2-eq	9.56E-01	1.03E+00	9.63E-01
Climate change- Land use and LU change	kg CO_2-eq	5.41E-04	5.48E-04	5.42E-04
Eutrophication, marine	kg N-eq	7.90E-04	8.15E-04	7.92E-04
Photochemical ozone formation	kg NMVOC-eq	5.88E-03	6.07E-03	5.90E-03
Eutrophication, freshwater	kg P-eq	7.98E-05	7.99E-05	7.98E-05
Resource use, minerals and metals	kg Sb-eq	2.65E-06	2.69E-06	2.65E-06
Water use	m^3 deprivation	3.94E-01	6.19E-01	7.63E-01
Resource use, fossils	MJ	1.62E+01	1.74E+01	1.63E+01
Acidification	mol H^+-eq	4.43E-03	4.50E-03	4.43E-03
Eutrophication, terrestrial	mol N-eq	8.72E-03	9.00E-03	8.75E-03
Land use	Pt	2.21E+00	2.24E+00	2.22E+00

Table S2. The cost calculation of hybrid process.

Total Annual Cost:<br>$\text{TAC} = \frac{TIC}{Years\ of\ amortization} + \text{TOC}$ (S4)
Distillation column and heat exchanger:<br>The Douglas cost equations [2] in euro (€) were employed to compute the IC estimate for the rectification column. Equations S5-S7 were then utilized to determine the IC for the external and internal components of the column, as well as for the heat exchangers. These equations incorporated inflation adjustments through the M&S index. Equation S8 was applied to establish the surface area of the heat exchangers. In the assessment of the OC, both the annual heating cost of the reboiler and the annual cooling water costs of the condenser were factored in Equation S9.<br>$\text{IC}_{\text{column outside}} = \left(\frac{\text{M\&S}}{280}\right) * 101.9 * D_c^{1.066} * \text{H}^{0.82} * (2.18 + \text{F}_\text{c})$ (S5)<br>$\text{IC}_{\text{column inside}} = \left(\frac{\text{M\&S}}{280}\right) * 4.7 * D_c^{1.55} * \text{H} * \text{F}_\text{c}$ (S6)<br>$\text{IC}_{\text{heat exchanger}} = \left(\frac{\text{M\&S}}{280}\right) * 101.3 * \text{A}^{0.65} * (2.29 + \text{F}_\text{c})$ (S7)<br>$\text{Q}=\text{k}*\text{A}*\Delta\text{T}$ (S8)<br>$\text{OC}_{\text{heat exchanger}} = Price_{utility} * Q_{heat\ exchanger}$ (S9)<br>Where:<br>M&S represents the Marshall & Swift equipment cost index, which, in this study, has a value of 1,773.4 [-].<br>ΔT is considered as the logarithmic temperature difference [K].<br>A represents the total heat transfer area [m^2].<br>D_c denotes the diameter of the column [m].<br>F_c stands for the constant [-]. For the external surface of the stainless-steel column, F_c equals 2.25; for the internal surface, F_c is 12.6; and for the heat exchangers, F_c is 5.1.<br>H signifies the column height [m], with a tray spacing of 0.6096 m.<br>k denotes the heat transfer coefficient [W/(m^2.K)].<br>Q stands for heat transfer [W].<br>$Price_{utility}$ represents the price of the cooling or heating utility used [€/GJ], in case of the cooling water is 0.34 €/GJ and the pressure steam is 7.42 €/GJ [3].<br>$Q_{heat\ exchanger}$ signifies the annual value of condenser or reboiler [GJ].

Pervaporation membrane:

The cost data for the pervaporation membrane is derived from González et al. [4] and is expressed through the following equations, which are grounded in 2007 price levels. The conversion from USD to Euro occurs at an identical exchange rate. Specifically, the pervaporation membrane is priced at 1,063 €/m^2, while the replacement cost per square meter is 200 €/m^2.

$$IC_{PV} = f_{Lang} * Price_{PV} * A_{PV,total} \quad (S10)$$

$$OC_{PV} = \frac{Price_{replacement} * A_{PV,total}}{t_{PV}} \quad (S11)$$

Where:

$A_{PV,total}$ denotes the total required PV membrane area [m^2].

f_{Lang} represents the Lang factor [-], which, in this instance, equals 3.36.

$Price_{PV}$ stands for the price per unit area of the PV membrane [€/m^2].

$Price_{reple}$ represents the yearly replacement cost of the membrane per unit area [€/year.m^2].

t_{PV} signifies the PV membrane's expected lifetime [year].

Table S3. TIC, TOC, TAC of the distillation columns of the investigated hybrid methods.

	n-propanol-water binary mixture				
	D+HPV+D		D+HPV+D+HI		D+HPV
	1st Dis.	2nd Dis.	1st Dis.	2nd Dis.	
IC of column outside [€]	148293	97362	148293	121173	148686
IC of column inside [€]	49603	28434	49603	39083	49794
IC of condenser [€]	10837	7742	10837	10624	10863
IC of reboiler [€]	45838	20860	31020	28266	45907
OC of cooling [€/year]	895	533	895	868	898
OC of heating [€/year]	39364	12253	21587	19555	39455
TOC of distillation [€/year]	40259	12786	22482	20423	40353
TIC of distillation [€/year]	25457	15440	23975	19915	25525
TAC of distillation €/year]	65716	28226	46457	40337	65878

Table S4. TIC, TOC, TAC of the pervaporation membranes of the investigated hybrid methods.

	n-propanol-water binary mixture		
	D+HPV	D+HPV+D	D+HPV+D+HI
Pervaporation membrane area [m^2]	360	120	120
Lifetime [year]	2	2	2
Calculation time [year]	10	10	10
Membrane investment cost [€]	1285805	428602	428602
Membrane replacement cost [€]	72000	24000	24000
TIC of PV [€/year]	128580	42860	42860
TOC of PV [€/year]	36000	12000	12000
TAC of PV[€/year]	164580	54860	54860

Table S5. TIC, TOC, TAC of the heat exchangers of the investigated hybrid methods.

	n-propanol-water binary mixture		
	D+HPV	D+HPV+D	D+HPV+D+HI
IC of coolers [€/year]	1474	2868	2668
IC of heaters [€/year]	5690	7327	6586
OC of cooling [€/year]	2057	2704	2224
OC of heating [€/year]	44883	56470	45994
TOC of heat exchangers [€/year]	7165	10196	9254
TIC of heat exchangers [€/year]	46940	59173	48218
TAC of heat exchangers [€/year]	54104	69369	57472

References

[1] B. Szilagyi, A. J. Toth, Improvement of Component Flux Estimating Model for Pervaporation Processes, Membranes 2020, 10, 418. https://doi.org/10.3390/membranes10120418
[2] J. M. Douglas, Conceptual design of chemical processes, McGraw-Hill, New York, 1988.
[3] K. Wang, L. Xin, Y. Zhang, J. Qi, Z. Zhu, Y. Wang, L. Zhong, P. Cui, Sustainable and efficient process design for wastewater recovery of cyclohexane/isopropyl alcohol azeotrope by extractive distillation based on multi-objective genetic algorithm optimization, Chemical Engineering Research and Design, 2024, 201, 593−602, https://doi.org/10.1016/j.cherd.2023.12.004
[4] B. González, I. Ortiz, Modelling and simulation of a hybrid process (pervaporation–distillation) for the separation of azeotropic mixtures of alcohol–ether, Journal of Chemical Technology & Biotechnology, 77, 2002, 29−42. https://doi.org/10.1002/jctb.526
